# Assessment of dementia risk scores in predicting mild cognitive impairment: A comparison of CogDrisk, CAIDE, LIBRA, and ANU-ADRI

**DOI:** 10.1016/j.tjpad.2025.100324

**Published:** 2025-08-12

**Authors:** Md Hamidul Huque, Kaarin J. Anstey

**Affiliations:** a UNSW Ageing Futures Institute, University of New South Wales, Australia; b School of Psychology, University of New South Wales, Australia; c Brain Health and Dementia Centre, Neuroscience Research Australia, Australia

**Keywords:** Risk prevention, Early prevention, Dementia risk factors, Risk prediction

## Abstract

**Background:**

Given the lack of widely accessible dementia treatments, identifying individuals at high risk of dementia is vital for prevention. No prior study has compared multiple validated dementia risk tools for predicting mild cognitive impairment (MCI) across multiple datasets. We assess the performance of the CogDrisk, ANU-ADRI, CAIDE, and LIBRA in predicting MCI.

**Method:**

Data were obtained from the ARIC, Whitehall II, and PATH Through Life cohorts. Participants without dementia or MCI at baseline were included. Risk scores were computed using available risk factors and analysed using logistic regression, with Area Under the Curve (AUC) estimates. Multiple imputation was used to evaluate the impact of missing data.

**Results:**

The ARIC (*n* = 5778), Whitehall II (*n* = 6387), and PATH (*n* = 2115) cohorts had mean baseline ages of 51.9, 55.8, and 62.5 years, with follow-ups of 28.2, 15.7, and 11.2 years, respectively. AUCs for MCI prediction were generally similar across tools and datasets. Dementia prevalence following MCI was highest in ARIC (23.6%), followed by Whitehall II (14.1%) and PATH (7.0%). In ARIC, CogDrisk showed slightly better AUCs for predicting MCI cases that progressed to dementia. Whitehall II and PATH showed mixed results, with wider confidence intervals for progressing MCI cases, and higher AUCs for non-progressing MCI cases using CogDrisk and ANU-ADRI. All tools performed consistently when predicting dementia without prior MCI.

**Discussion:**

Dementia risk scores demonstrated comparable performance of MCI prediction and are more sensitive for identifying cases that progress to dementia, supporting their greater utility for informing risk reduction strategies.

## 1 Introduction

Dementia is a leading cause of disability and dependency among older adults, currently affecting more than 55 million people worldwide [1]. As the global population ages, the number of the affected individuals is expected to triple by 2050, reaching an estimated 139 million patients globally [2]. To date, there is no curative or widely accessible treatment for dementia. As a result, prevention strategies that aim to improve and to raise awareness of dementia risk factors among patients and health practitioners are urgently needed [3].

Mild cognitive impairment (MCI) is an early stage of cognitive decline that can lead to dementia. Research shows that the prevention of MCI and dementia can be achieved by better management of cardiovascular risk and the adoption of healthier lifestyle choices [3,4]. The *Lancet* commission report identified 14 modifiable risk factors, which, with appropriate interventions, could prevent nearly half of dementia cases in the population [5]. Therefore, identifying and managing these risk factors is critically important, as it may help delay the onset of dementia [6].

Dementia risk assessment, through the calculation of dementia risk scores, a weighted composite of risk factors that reflects the likelihood of an individual developing dementia, is currently being used as a prevention strategy to improve risk perception among patients and clinicians. Several validated risk scores including ANU-ADRI [7], LIBRA [8], CAIDE [9] and CogDrisk [10] have been developed in the literature using data from older adults to predict dementia risk. Among these, CAIDE [9] was developed nearly two decades ago using midlife population from a single cohort, however it demonstrated only moderate predictive accuracy when validated in independent cohort data [11,12]. The CogDrisk incorporates contemporary risk factors and has shown superior predictive performance for dementia in older populations [11,13].

While ANU-ADRI [14] and LIBRA [8] scores have been validated for risk prediction of MCI, no such studies are available in the literature that validate the use the CogDrisk score for this purpose. No prior study has compared multiple dementia risk assessment tools in their accuracy for predicting MCI and no study has evaluated this question on multiple datasets.

Therefore, the aim of our study is to evaluate the performance of CogDrisk, ANU-ADRI, LIBRA and CAIDE dementia risk scores for predicting MCI using secondary data from longitudinal studies conducted in the USA, UK and Australia. All these dataset include clinical assessments of MCI using validated methods.

## 2 Methods

We aimed to study the performance of the above risk assessment tools using data from three longitudinal studies: the Atherosclerosis Risk in Communities Study (ARIC) study, the Whitehall II study, and the Personality and Total Health through life (PATH) study.

### 2.1 The atherosclerosis risk in communities (ARIC)

The ARIC cohort study was conducted in four geographically diverse U.S. communities and was initially designed to identify risk factors for subclinical atherosclerosis. As participants aged, the study’s focus expanded to identify risk factors for heart diseases and dementia. The cohort included 15,792 participants aged 45 to 66 years at their baseline (Exam 1: 1987–89) [15]. While dementia typically manifests in later life, many of its key modifiable risk factors such as hypertension, obesity, diabetes, smoking, and high LDL cholesterol begin to emerge in mid‑life. Also the age distribution of ARIC Exam 1 correspond to the middle aged population aligned with the age distribution of other two cohorts, hence we used Exam 1 as baseline in the dataset. While the study is still ongoing, follow-up data are available up to Exam 7 (2018–19). Dementia and MCI were first assessed during Exam 5 (2011–13). For the current analysis, we therefore included 5778 participants who had valid MCI status (in wave either 5, 6 or 7), free of dementia or MCI at wave 5, and have non-missing education status at baseline. MCI and Dementia was diagnosed using an algorithm and ICD-9 codes, following the Diagnostic and Statistical Manual of Mental Disorders (DSM-III-R) criteria [16]. All the participants included in our analysis from the ARIC dataset had follow-up period of approximately 29 years (from Exam 1 to Exam 7). The dataset was obtained via BioLINCC (Application # 13,630).

### 2.2 The Whitehall II

The Whitehall II is a longitudinal cohort of 10,308 British Civil servants aged 35 to 55 at baseline (1987–1989), designed to investigate the social determinants of health [17]. Twelve waves of data collections are available with wave 12 conducted in 2015–2016. To align with the ARIC cohort in terms of age distribution, we selected Wave 5 (1997-99) as the baseline. Furthermore, Wave 5 includes most of the variables required for calculating risk scores. In total, 6387 participants aged 45 to 65 and free of MCI or dementia at wave 5 were included in the analysis. Probable MCI was defined based on MMSE score between 19 and 23 [18,19] at any follow-up assessment (wave 7, 9, 11 or 12) and will hereforth be referred to as MCI for the purpose of this study. In this cohort, dementia was defined based on self-reported dementia diagnosis during follow-up. The datasets were obtained and analysed through the Dementias Platform UK (Application # 704).

### 2.3 The personality and total health (PATH) through life

The PATH study is an ongoing, population-based, Australian longitudinal cohort comprising approximately 7500 participants across three life stages: early life (*n* = 2404; age 20–24), midlife (*n* = 2530; age 40–44), and late life (*n* = 2509; age 60–64) at baseline (2001–2002) [20]. The detailed study methodology has been reported elsewhere [20]. In the current analysis, we only considered late-life cohorts participants (*n* = 2115) who were free of MCI at baseline and had non-missing data on education. MCI and dementia was diagnosed according to DSM-IV (Diagnostic and Statistical Manual of Mental Disorders, Fourth Edition) criteria [21]. We considered up to 12 years of follow-up data for this analysis (Wave1 to Wave 4).

### 2.4 Prediction tools included for analysis

The specific predictive tools/models applied to the above cohorts are briefly described below:

### 2.5 Cognitive health and dementia risk Assessment (CogDrisk)

The CogDrisk was developed to assess dementia risk using 17 risk and protective factors, including age, sex, low education, midlife obesity, diabetes, depression, midlife high cholesterol, traumatic brain injury (TBI), smoking, loneliness, physical inactivity, cognitive activity, fish intake, hypertension, stroke, atrial fibrillation and insomnia. Based on systematic reviews and meta-analyses, risk ratios were obtained for each factor and later converted into point values for risk scoring. Previous research indicates that CogDrisk has moderate to good predictive power for dementia [11,13] and has the highest number of risk factors included in the WHO Guidelines for Risk Reduction of Cognitive Decline and Dementia [12].

### 2.6 The Australian national university Alzheimer’s disease risk index (ANU-ADRI)

The ANU-ADRI was developed for use in public health settings and is designed to be completed without the need for clinical assessment. It evaluates 11 risk factors and 4 protective factors associated with Alzheimer’s disease (AD). The tool has been externally validated across multiple cohort studies for predicting AD [22], dementia [14], and MCI [21], and has also been linked to Cognitive decline in randomized control trials [23]. Like CogDrisk, ANU-ADRI identifies relevant risk and protective factors for AD through comprehensive evidence syntheses, with relative risks for each factor were from existing meta-analyses or relevant cohort studies, and converted into a point-based scoring system.

### 2.7 LIfestyle for BRAin health (LIBRA)

The LIBRA dementia risk tool was developed through a literature review and Delphi consensus process and risk weights were derived [8]. It focuses exclusively on modifiable risk factors and therefore excludes age and sex from risk score calculation. Similar to CogDrisk and ANU-ADRI, LIBRA designed for use in population-based and public health contexts without requiring clinical assessments. LIBRA scores have been shown to be associated with both late-life dementia and MCI [24].

### 2.8 The cardiovascular risk factors, aging, and incidence of dementia (CAIDE)

The CAIDE score is a midlife risk prediction tool developed using data from the CAIDE cohort, a Finnish population based cohort aged 39–64 years [9]. It estimates 20-year dementia risk based on the individual’s midlife risk factors profile using logistic regression. Regression coefficients were converted into a point-based score. CAIDE has been found to be predictive of MCI among Asian, Latinx, and non-Latinx White participants, but not among Black participants [25].

### 2.9 Statistical analysis

For each cohort included in the analysis, risk factor data were obtained either from the baseline assessment or, when unavailable, from the nearest available follow-up, under the assumption that these characteristics remained stable over time. A detailed definition of risk and protective factors are available in Supplementary Table S1. The total risk score for each participant was calculated by summing the point values, defined according to the original risk tool development, for all available risk factors at baseline or from the nearest available follow-up within each cohort. The discriminative ability of the risk scores to identify individuals at risk of MCI was evaluated by calculating the area under the receiver operating characteristic curve (AUC) along with 95 % confidence intervals (CIs) [26].

Since age and sex are among the most influential risk factors for dementia, and LIBRA risk score does not include age, sex or education, we also conducted comparisons of AUCs excluding these variables from the other risk scores for consistency. A sensitivity analysis was also conducted to assess the performance of risk scores in predicting MCI cases that later progressed to dementia compared to those that did not.

To account for the potential impact of missing data on MCI risk estimation, we use (i) complete case analysis (i.e., excluding participants with missing data in risk factor) in risk score calculation, and (ii)multiple imputation using a multivariate normal model, generating 20 imputed datasets [27]. The imputation models included relevant covariates to ensure consistency with the subsequent analysis models. All statistical analyses were performed using Stata version 16.1 (StataCorp, College Station, TX) between December 2024 and March 2025.

## 3 Results

### 3.1 Description of the study cohorts

Table 1 presents the distribution of risk and protective factors for assessing dementia risk in the ARIC, the Whitehall, and the PATH study cohorts. A total of 5778, 6380, and 2386 participants are available in the ARIC, Whitehall and the PATH cohorts, respectively. Participants are followed up for a mean (standard deviation) of 28.2 (3.1), 15.7 (4.1), and 11.2 (2.7) years, respectively.

**Table 1:** Descriptive statistics for the three evaluation cohorts in terms of risk and protective factors used in various prediction models.

	ARIC Study		Whitehall Study		PATH Study	
Covariates	Male n=2422 (%)	Female n=3356 (%)	Male n=4570 (%)	Female n= 1817 (%)	Male n=1081 (%)	Female n= 1034 (%)
**Age, years (mean, sd)**	52.1 (5.1)	51.7 (5.0)	55.7 (6.0)	56.1 (6.0)	62.4 (1.5)	62.5 (1.5)
**Age group**						
45-49	898 (37.1)	1376 (41.0)	989 (21.6)	355 (19.5)	NA	NA
50-54	756 (31.2)	1023 (30.5)	1339 (29.3)	488 (26.9)		
55-59	523 (21.6)	648 (19.3)	926 (20.3)	400 (22.0)		
60-64	238 (9.8)	304 (9.1)	963 (21.1)	429 (23.6)	968 (89.5)	901 (87.1)
65-69	7 (0.3)	5 (0.1)	353 (7.7)	145 (8.0)	113 (10.5)	133 (12.9)
**Education**						
0-<8 years	291 (12.0)	443 (13.2)	141 (3.1)	61 (3.4)	13 (1.2)	29 (2.8)
8-11 years	914 (37.7)	1552 (46.2)	572 (12.5)	471 (25.9)	182 (16.8)	268 (25.9)
>11 years	1217 (50.2)	1361 (40.6)	3857 (84.4)	1285 (70.7)	886 (82.0)	737 (71.3)
**Obesity**						
Underweight	4 (0.2)	26 (0.8)	22 (0.5)	27 (1.5)	4 (0.4)	11 (1.1)
Normal weight	675 (27.9)	1438 (42.8)	1951 (42.7)	802 (44.1)	334 (30.9)	416 (40.2)
Overweight	1262 (52.1)	1058 (31.5)	2005 (43.9)	614 (33.8)	499 (46.2)	305 (29.5)
Obese	480 (19.8)	834 (24.9)	483 (10.6)	334 (18.4)	156 (14.4)	195 (18.9)
Missing	1 (0.0)	0 (0.0)	109 (2.4)	40 (2.2)	88 (8.1)	107 (10.3)
**Diabetes**						
Yes	687 (28.4)	877 (26.1)	196 (4.3)	95 (5.2)	82 (7.6)	55 (5.3)
Missing	119 (4.9)	229 (6.8)	8 (0.2)	5 (0.3)	0 (0.0)	0 (0.0)
**Depression**						
Yes	40 (1.7)	123 (3.7)	250 (5.5)	161 (8.9)	9 (0.8)	11 (1.1)
Missing	125 (5.2)	223 (6.6)	385 (8.4)	194 (10.7)	1 (0.1)	5 (0.5)
**High cholesterol**						
Yes	472 (19.5)	728 (21.7)	1149 (25.1)	514 (28.3)	278 (25.7)	214 (20.7)
Missing	21 (0.9)	49 (1.5)	96 (2.1)	33 (1.8)	0 (0.0)	0 (0.0)
**Traumatic Brain Injury (TBI)**						
Yes	344 (14.2)	300 (8.9)	NA		83 (7.7)	29 (2.8)
Missing	21 (0.9)	22 (0.7)			0 (0.0)	0 (0.0)
**Smoking status**						
Never smoker	868 (35.8)	1916 (57.1)	2228 (48.8)	1010 (55.6)	471 (43.6)	667 (64.5)
Former smoker	1123 (46.4)	833 (24.8)	1800 (39.4)	517 (28.5)	493 (45.6)	278 (26.9)
Current smoker	431 (17.8)	604 (18.0)	390 (8.5)	211 (11.6)	117 (10.8)	89 (8.6)
Missing	0 (0.0)	3 (0.1)	152 (3.3)	79 (4.4)	0 (0.0)	0 (0.0)
**Alcohol drink**						
Abstainer	1066 (44.0)	2290 (68.2)	NR		90 (8.3)	194 (18.8)
Low to moderate	1089 (45.0)	981 (29.2)			749 (69.3)	785 (75.9)
High	260 (10.7)	77 (2.3)			241 (22.3)	52 (5.0)
Missing	7 (0.3)	8 (0.2)			1 (0.1)	3 (0.3)
**Loneliness**						
Yes	NA		989 (21.6)	580 (31.9)	68 (6.3)	93 (9.0)
Missing			70 (1.5)	49 (2.7)	12 (1.1)	10 (1.0)
**Physical activity**						
Less than sufficient	641 (26.5)	1228 (36.6)	622 (13.6)	543 (29.9)	411 (38.0)	552 (53.4)
Missing	215 (8.9)	385 (11.5)	51 (1.1)	28 (1.5)	134 (12.4)	122 (11.8)
**Cognitive activity**						
Low	NA		NA		240 (22.2)	270 (26.1)
Mid					397 (36.7)	434 (42.0)
High					440 (40.7)	324 (31.3)
Missing					4 (0.4)	6 (0.6)
**Fish serve, mean (sd)**	1.67 (2.0)	1.68 (2.0)	1.94 (1.7)	2.39 (2.2)	0.35 (0.4)	0.45 (0.5)
**Hypertension**						
Yes	561 (23.2)	809 (24.1)	944 (20.7)	491 (27.0)	732 (67.7)	597 (57.7)
Missing	17 (0.7)	14 (0.4)	19 (0.4)	10 (0.6)	12 (1.1)	14 (1.4)
**Stroke**						
Yes	92 (3.8)	101 (3.0)	24 (0.5)	5 (0.3)	41 (3.8)	41 (4.0)
Missing	77 (3.2)	134 (4.0)	0 (0.0)	0 (0.0)	0 (0.0)	0 (0.0)
**Coronary heart disease**						
Yes	555 (22.9)	265 (7.9)	NA		NA	
Missing	105 (4.3)	195 (5.8)				
**Kidney disease**						
Yes	22 (0.9)	41 (1.2)	NA		NA	
Missing	22 (0.9)	30 (0.9)				
**Atrial Fibrillation**						
Yes	662 (27.3)	800 (23.8)	NA		NA	
Missing	52 (2.1)	99 (2.9)				
**Sleep problem**						
Yes	NA		107 (2.3)	42 (2.3)	109 (10.1)	135 (13.1)
Missing			65 (1.4)	39 (2.2)	1 (0.1)	4 (0.4)
**Mild cognitive impairment (MCI)**						
**Yes**	970 (40.0)	1070 (31.9)	104 (2.3)	52 (2.9)	100 (9.3)	72 (7.0)
**Dementia**						
**Yes**	380 (15.7)	511 (15.2)	43 (0.9)	18 (1.0)	24 (2.2)	21 (2.0)
**Missing**	0 (0.0)	0 (0.0)	0 (0.0)	0 (0.0)	269 (24.9)	301 (29.1)

NA: Not Available; NR: Not requested during data access.

Although the majority of participants were middle-aged at baseline (99.8 % in ARIC, 92.8 % in Whitehall II, and 89.4 % in PATH), the PATH cohort participants are slightly older compared to other two cohorts and have similar distribution of males and females. On the contrary, the ARIC participants are predominantly female (58 %) and Whitehall II participants are mostly male (72 %).

All three cohorts have information on age, sex, education, obesity, diabetes, depression, high cholesterol, and smoking status, physical activity, fish intake, hypertension, and stroke. However, the ARIC cohort did not collect information on loneliness, cognitive activity, and sleep problems, while both the Whitehall II and PATH cohorts lacked information on coronary heart disease and kidney disease. Additionally, data on traumatic brain injury (TBI) were not available in the Whitehall cohort.

In terms of risk and protective factors distributions, higher proportions of males compared with females had completed tertiary degree in all three cohorts. In all cohorts, males were also more likely to be smokers and less physically active, but less likely to be obese compared to females. The prevalence of MCI was highest in the ARIC cohort (35.3 %), followed by PATH (8.1 %) and the Whitehall II study (2.4 %).

### 3.2 Comparison of predictive ability of CogDrisk, ANU-ADRI, CAIDE, and LIBRA risk scores for predicting MCI in three cohorts

Table 2 presents the odds ratios (ORs, with 95 % CIs) and predictive performance (AUCs, with 95 % CIs) of four dementia risk scores: CogDrisk, ANU-ADRI, CAIDE, and LIBRA, across the ARIC, Whitehall II, and PATH cohorts. Analyses were performed using both complete case data and datasets following multiple imputation.

**Table 2:** CogDrisk score in predicting MCI using logistic regression model.

ARIC data: Complete case analysis: Male=1992, Female=2619, Total=4611								
	CogDrisk		ANU-ADRI		CAIDE		LIBRA	
Sex	OR (95 % CI)	AUC (95 % CI)	OR (95 % CI)	AUC (95 % CI)	OR (95 % CI)	AUC (95 % CI)	OR (95 % CI)	AUC (95 % CI)
Male	1.04 (1.02, 1.07)	0.54 (0.51, 0.56)	1.01 (1.00, 1.03)	0.52 (0.50, 0.55)	1.11 (1.06, 1.16)	0.56 (0.53, 0.58)	1.05 (0.99, 1.11)	0.52 (0.49, 0.55)
Female	1.04 (1.02, 1.06)	0.55 (0.52, 0.57)	1.01 (1.00, 1.03)	0.52 (0.50, 0.55)	1.10 (1.04, 1.13)	0.55 (0.53, 0.57)	1.03 (0.99, 1.09)	0.52 (0.49, 0.54)
Overall	1.04 (1.02, 1.05)	0.54 (0.52, 0.55)	1.01 (1.00, 1.02)	0.52 (0.50, 0.54)	1.11 (1.08, 0.14)	0.57 (0.55, 0.58)	1.05 (1.01, 1.09)	0.52 (0.50, 0.54)
ARIC data: Analysis of Multiple imputed dataset, Male=2422, Female=3356, Total=5778								
Male	1.03 (1.00, 1.07)	0.52 (0.50, 0.55)	1.00 (0.98, 1.02)	0.51 (0.48, 0.53)	1.09 (1.04, 1.14)	0.54 (0.52, 0.57)	1.08 (1.02, 1.14)	0.53 (0.51, 0.55)
Female	1.05 (1.02, 1.07)	0.54 (0.52, 0.56)	1.01 (0.99, 1.03)	0.51 (0.49, 0.53)	1.07 (1.03, 1.12)	0.53 (0.51, 0.55)	1.09 (1.04, 1.14)	0.53 (0.51, 0.55)
Overall	1.07 (1.04, 1.09)	0.53 (0.51, 0.54)	1.00 (0.99, 1.02)	0.50 (0.49, 0.52)	1.11 (1.07, 1.14)	0.55 (0.54, 0.57)	1.08 (1.04, 1.12)	0.53 (0.51, 0.54)
Whitehall data: Complete case analysis: Male=3172, Female=1173, Total=4345								
Male	1.11 (1.02, 1.21)	0.56 (0.50, 0.63)	0.99 (0.94, 1.05)	0.49 (0.42, 0.55)	0.94 (0.84, 1.07)	0.48 (0.41, 0.54)	0.91 (0.75, 1.11)	0.47 (0.41, 0.53)
Female	1.05 (0.93, 1.19)	0.55 (0.43, 0.67)	1.08 (1.00, 1.17)	0.59 (0.49, 0.69)	1.04 (0.89, 1.21)	0.56 (0.46, 0.67)	0.97 (0.75, 1.25)	0.52 (0.41, 0.62)
Overall	1.09 (1.02, 1.17)	0.56 (0.50, 0.62)	1.02 (0.97, 1.07)	0.52 (0.46, 0.57)	0.97 (0.89, 1.07)	0.50 (0.44, 0.55)	0.93 (0.80, 1.09)	0.48 (0.43, 0.54)
Whitehall data: Analysis of Multiple imputed dataset, Male=4570, Female=1817, Total=6387								
Male	1.12 (1.05, 1.19)	0.58 (0.53, 0.64)	1.02 (0.98, 1.06)	0.52 (0.46, 0.57)	1.01 (0.93, 1.10)	0.53 (0.47, 0.58)	1.04 (0.91, 1.18)	0.51 (0.45, 0.56)
Female	1.08 (1.00, 1.17)	0.56 (0.48, 0.65)	1.09 (1.03, 1.14)	0.61 (0.54, 0.69)	1.10 (0.99, 0.121)	0.60 (0.52, 0.67)	1.08 (0.92, 1.27)	0.53 (0.45, 0.61)
Overall	1.11 (1.05, 1.16)	0.58 (0.53, 0.62)	1.05 (1.01, 1.08)	0.55 (0.50, 0.60)	1.04 (0.97, 1.11)	0.55 (0.50, 0.59)	1.06 (0.96, 1.17)	0.52 (0.47, 0.56)
PATH data: Complete case analysis: Male=712, Female=673, Total=1385								
Male	1.04 (0.98, 1.11)	0.55 (0.48, 0.62)	1.07 (1.02, 1.11)	0.61 (0.54, 0.67)	1.09 (0.94, 1.27)	0.53 (0.47, 0.60)	1.07 (0.92, 1.25)	0.53 (0.45, 0.60)
Female	1.05 (0.99, 1.12)	0.55 (0.47, 0.62)	1.06 (1.01, 1.11)	0.58 (0.50, 0.66)	1.11 (0.93, 1.31)	0.54 (0.46, 0.62)	1.08 (0.90, 1.30)	0.53 (0.45, 0.62)
Overall	1.04 (1.00, 1.09)	0.54 (0.49, 0.60)	1.06 (1.03, 1.09)	0.59 (0.54, 0.64)	1.12 (1.00, 1.25)	0.55 (0.50, 0.60)	1.07 (0.95, 1.21)	0.53 (0.47,0.58)
PATH data: Analysis of Multiple imputed dataset, Male=1081, Female=1034, Total=2115								
Male	1.04 (0.99, 1.10)	0.54 (0.48, 0.60)	1.06 (1.01, 1.10)	0.57 (0.51, 0.63)	1.07 (0.94, 1.21)	0.52 (0.46, 0.58)	1.07 (0.93, 1.24)	0.53 (0.47, 0.60)
Female	1.06 (1.00, 1.12)	0.57 (0.50, 0.64)	1.06 (1.01, 1.11)	0.59 (0.52, 0.66)	1.09 (0.94, 1.26)	0.54 (0.47, 0.61)	1.10 (0.93, 1.29)	0.54 (0.47, 0.61)
Overall	1.05 (1.01, 1.09)	0.55 (0.50, 0.60)	1.06 (1.02, 1.09)	0.58 (0.54, 0.63)	1.10 (1.00, 1.20)	0.55 (0.50, 0.59)	1.08 (0.97, 1.20)	0.53 (0.49, 0.58)

In the ARIC dataset, the CAIDE risk score produced the highest ORs for predicting MCI in both complete case and imputed analyses. The CogDrisk and LIBRA scores yielded similar, though slightly lower, ORs in the complete case analysis. However, in the imputed dataset, the predicted performance are comparable across CogDrisk: and LIBRA, whereas ANU-ADRI resulted in slightly lower AUC. In the Whitehall II dataset, the complete case analysis produced slightly lower AUCs compared to the multiple imputed analysis across all risk scores. In the imputed dataset, the AUCs were generally similar for CogDrisk, ANU-ADRI, CAIDE, and LIBRA. In the PATH dataset, the CAIDE score also yielded the highest ORs for predicting mild cognitive impairment (MCI), while the ANU-ADRI score produced the highest AUC followed by CogDrisk, and CAIDE. Across all three cohorts, the CogDrisk score demonstrated consistent AUCs.

### 3.3 Comparison of predictive ability of risk scores for predicting MCI without age, sex and education

To ensure consistency in evaluating predictive performance across risk scores, we recalculated the CogDrisk, ANU-ADRI, CAIDE, and LIBRA scores excluding age, sex, and education. This was due to the demographic profile of the study cohorts, which primarily comprised midlife populations (aged 40–64 years). Notably, the CogDrisk, ANU-ADRI, and LIBRA risk scores do not incorporate weighting for age or sex in this age range, and the LIBRA score further omits educational attainment from its algorithm. The results of this reduced model are presented in Table 3. In general, all four risk scores resulted in similar AUCs across the three cohorts. Additionally, similar predictive ability was observed between males and females across all datasets and risk scores. Complete case analysis produced similar AUCs as of multiple imputed data.

**Table 3:** CogDrisk score in predicting MCI using logistic regression model without age, sex and education estimates.

ARIC data: Complete case analysis: Male=1992, Female=2619, Total=4611								
	CogDrisk		ANU-ADRI		CAIDE		LIBRA	
Sex	OR (95 % CI)	AUC (95 % CI)	OR (95 % CI)	AUC (95 % CI)	OR (95 % CI)	AUC (95 % CI)	OR (95 % CI)	AUC (95 % CI)
Male	1.06 (1.02, 1.09)	0.54 (0.51, 0.57)	1.02 (1.00, 1.04)	0.52 (0.50, 0.55)	1.05 (0.99, 1.11)	0.52 (0.49, 0.54)	1.04 (0.98, 1.10)	0.52 (0.49, 0.54)
Female	1.05 (1.03, 1.08)	0.55 (0.52, 0.57)	1.02 (1.00, 1.03)	0.52 (0.49, 0.55)	1.03 (0.98, 1.08)	0.51 (0.49, 0.54)	1.03 (0.98, 1.09)	0.52 (0.49, 0.54)
Overall	1.05 (1.03, 1.07)	0.54 (0.52, 0.56)	1.02 (1.00, 1.03)	0.52 (0.50, 0.54)	1.03 (0.99, 1.07)	0.51 (0.50, 0.53)	1.04 (1.00, 1.08)	0.52 (0.50, 0.54)
ARIC data: Analysis of Multiple imputed dataset, Male=2422, Female=3356, Total=5778								
Male	1.06 (1.02, 1.10)	0.53 (0.50, 0.55)	1.00 (0.97, 1.02)	0.50 (0.48, 0.53)	1.01 (0.94, 1.08)	0.50 (0.48, 0.53)	1.08 (1.02, 1.14)	0.53 (0.51, 0.55)
Female	1.07 (1.04, 1.10)	0.55 (0.52, 0.57)	1.02 (0.99, 1.04)	0.52 (0.50, 0.54)	0.94 (0.89, 1.00)	0.52 (0.50, 0.54)	1.09 (1.04, 1.14)	0.53 (0.51, 0.55)
Overall	1.06 (1.04, 1.09)	0.54 (0.52, 0.55)	1.01 (0.99, 1.03)	0.51 (0.49, 0.52)	0.98 (0.94, 1.03)	0.51 (0.49, 0.52)	1.08 (1.04, 1.12)	0.53 (0.51, 0.54)
Whitehall data: Complete case analysis: Male=3172 Female=1173, Total=4345								
Male	0.91 (0.82, 1.00)	0.41 (0.34, 0.48)	0.94 (0.89, 1.01)	0.44 (0.38, 0.50)	1.00 (0.85, 1.17)	0.49 (0.42, 0.56)	0.91 (0.75, 1.11)	0.47 (0.41, 0.53)
Female	1.02 (0.89, 1.17)	0.53 (0.42, 0.65)	1.07 (0.98, 1.17)	0.57 (0.46, 0.68)	1.16 (0.95, 1.44)	0.60 (0.49, 0.70)	0.97 (0.75, 1.25)	0.52 (0.41, 0.62)
Overall	0.94 (0.87, 1.02)	0.45 (0.38, 0.51)	0.98 (0.93, 1.03)	0.48 (0.42, 0.53)	1.05 (0.93, 1.20)	0.52 (0.46, 0.58)	0.93 (0.80, 1.09)	0.48 (0.43, 0.54)
Whitehall data: Analysis of Multiple imputed dataset, Male=4570, Female=1817, Total=6387								
Male	0.98 (0.92, 1.06)	0.53 (0.47, 0.59)	0.98 (0.94, 1.03)	0.52 (0.46, 0.58)	1.12 (1.00, 1.26)	0.54 (0.49, 0.60)	1.04 (0.91, 1.18)	0.51 (0.45, 0.56)
Female	1.02 (0.93, 1.12)	0.51 (0.42, 0.59)	1.06 (1.00, 1.12)	0.58 (0.50, 0.66)	1.15 (0.99, 1.33)	0.59 (0.52, 0.67)	1.08 (0.92, 1.27)	0.53 (0.45, 0.61)
Overall	1.00 (0.95, 1.06)	0.48 (0.43, 0.53)	1.02 (0.98, 1.05)	0.52 (0.50, 0.56)	1.14 (1.04, 1.25)	0.56 (0.51, 0.61)	1.06 (0.96, 1.17)	0.52 (0.47, 0.56)
PATH data: Complete case analysis: Male=712, Female=673, Total=1385								
Male	1.05 (0.98, 1.13)	0.55 (0.48, 0.62)	1.06 (1.01, 1.11)	0.59 (0.52, 0.66)	1.09 (0.94,1.27)	0.53 (0.47, 0.60)	1.07 (0.92, 1.25)	0.53 (0.45, 0.60)
Female	1.03 (0.96, 1.11)	0.53 (0.45, 0.61)	1.06 (1.01, 1.12)	0.58 (0.49, 0.66)	1.11 (0.93, 1.31)	0.54 (0.46, 0.62)	1.08 (0.90, 1.30)	0.53 (0.45, 0.62)
Overall	1.03 (0.98, 1.09)	0.53 (0.48, 0.59)	1.06 (1.02, 1.09)	0.58 (0.58, 0.63)	1.10 (0.98, 1.23)	0.54 (0.49, 0.59)	1.07 (0.95, 1.21)	0.53 (0.47,0.58)
PATH data: Analysis of Multiple imputed dataset, Male=1081, Female=1034, Total=2115								
Male	1.03 (0.97, 1.10)	0.53 (0.47, 0.59)	1.04 (0.99, 1.09)	0.54 (0.48, 0.60)	0.99 (0.87, 1.13)	0.50 (0.45, 0.56)	1.07 (0.93, 1.24)	0.53 (0.47, 0.60)
Female	1.04 (0.98, 1.12)	0.55 (0.48, 0.62)	1.08 (1.02, 1.15)	0.57 (0.50, 0.64)	1.05 (0.89, 1.23)	0.52 (0.45, 0.59)	1.10 (0.93, 1.29)	0.54 (0.47, 0.61)
Overall	1.03 (0.99, 1.08)	0.53 (0.49, 0.58)	1.06 (1.02, 1.10)	0.56 (0.51, 0.60)	1.02 (0.93, 1.13)	0.51 (0.47, 0.55)	1.08 (0.97, 1.20)	0.53 (0.49, 0.58)

### 3.4 Comparison of predictive ability of risk scores for predicting various subgroups of MCI/dementia

Table 4 presents the comparative performance of risk scores in predicting MCI (or probable MCI in the Whitehall study) cases that later progressed to dementia versus that did not. In the ARIC cohort, all risk scores showed higher AUCs for predicting MCI cases that later progressed to dementia compared to those that did not. CogDrisk achieved the highest AUC in this group and also showed a similar AUC for predicting dementia cases without a prior MCI diagnosis.

**Table 4:** Performance of risk scores for predicting various MCI and dementia subgroups including (i) MCI cases that later progressed to dementia, (ii) MCI cases that didn’t, and (iii) dementia cases without prior MCI diagnosis in all three cohorts.

		CogDrisk		ANU-ADRI		CAIDE		LIBRA	
ARIC Cohort (*n* = 5778)	n ( %)	Predicting Dementia AUC (95 %)]	Predicting MCI AUC (95 %)]	Predicting Dementia AUC (95 %)]	Predicting MCI AUC (95 %)]	Predicting Dementia AUC (95 %)]	Predicting MCI AUC (95 %)]	Predicting Dementia AUC (95 %)]	Predicting MCI AUC (95 %)]
No MCI and No dementia	3329 (57.7)	Ref	Ref	Ref	Ref	Ref	Ref	Ref	Ref
Dementia & MCI	482 (8.3)	0.61 (0.58, 0.64)	0.61 (0.58, 0.64)	0.54 (0.51, 0.57)	0.54 (0.51, 0.56)	0.59 (0.56, 0.62)	0.59 (0.56, 0.62)	0.56 (0.53, 0.59)	0.56 (0.53, 0.59)
Dementia without MCI	409 (7.1)	0.63 (0.61, 0.66)	NA	0.58 (0.54, 0.61)	NA	0.57 (0.55, 0.60)	NA	0.57 (0.54, 0.60)	NA
MCI without dementia	1558 (26.9)	NA	0.52 (0.51, 0.54)	NA	0.50 (0.48, 0.52)	NA	0.55 (0.53, 0.57)	NA	0.53 (0.51, 0.55)
PATH Cohort (*n* = 1545)									
No MCI and No dementia	1353 (87.6)	Ref	Ref	Ref	Ref	Ref	Ref	Ref	Ref
Dementia & MCI	11 (0.7)	0.53 (0.34, 0.72)	0.53 (0.34, 0.72)	0.52 (0.31, 0.73)	0.52 (0.31, 0.73)	0.54 (0.35, 0.72)	0.54 (0.35, 0.72)	0.57 (0.37, 0.77)	0.57 (0.37, 0.77)
Dementia without MCI	34 (2.2)	0.57 (0.47, 0.67)	NA	0.52 (0.41, 0.63)	NA	0.55 (0.45, 0.66)	NA	0.58 (0.48, 0.68)	NA
MCI without dementia	147 (9.5)	NA	0.56 (0.51, 0.61)	NA	0.59 (0.54, 0.64)	NA	0.55 (0.50, 0.60)	NA	0.55 (0.50, 0.60)
Whitehall II cohort (*n* = 6387)									
No MCI and No dementia	6192 (97.0)	Ref	Ref	Ref	Ref	Ref	Ref	Ref	Ref
Dementia & MCI	22 (0.3)	0.52 (0.40, 0.63)	0.52 (0.40, 0.63)	0.68 (0.60, 0.77)	0.68 (0.60, 0.77)	0.49 (0.39, 0.59)	0.49 (0.39, 0.59)	0.53 (0.41, 0.66)	0.53 (0.41, 0.66)
Dementia without MCI	39 (0.6)	0.51 (0.42, 0.60)	NA	0.61 (0.52, 0.71)	NA	0.56 (0.48, 0.64)	NA	0.51 (0.41, 0.60)	NA
MCI without dementia	134 (2.1)	NA	0.59 (0.53, 0.64)	NA	0.63 (0.54, 0.64)	NA	0.55 (0.51, 0.60)	NA	0.52 (0.47, 0.57)

In contrast, in the Whitehall II cohort, lower AUCs were observed for MCI cases that progressed to dementia compared to those that did not for both CogDrisk and CAIDE. ANU-ADRI, however, showed higher AUCs for predicting MCI cases that later developed dementia compared to non-progressing cases. LIBRA again produced similar AUCs across both groups. Among all tools, ANU-ADRI yielded the highest predictive performance for identifying dementia cases without prior MCI in this cohort.

In the PATH cohort, CogDrisk, CAIDE, and LIBRA exhibited comparable performance in predicting MCI cases that later progressed to dementia and MCI cases that did not progress. ANU-ADRI showed the highest AUC for non-progressing MCI cases and the lowest for dementia cases without prior MCI. Across all tools, AUCs for predicting MCI cases that progressed to dementia were similar but associated with wider confidence intervals in this dataset.

## 4 Discussion

We report the first ever comparison of multiple dementia risk assessment tools for predicting MCI using data from multiple cohort studies. This is also the first study to evaluate the more recently developed CogDrisk, in predicting MCI. Our findings indicate that the CogDrisk score demonstrated predictive performance comparable to the established tools, showing consistent discriminative ability across all three cohorts. Similar AUCs for predicting MCI based on modifiable risk factors have also been reported in previous studies [28]. Notably, the majority of participants in our cohorts were in midlife, and age- and sex-specific weights were not available for this population. Given that age and sex are well-established risk factors for dementia and play a significant role in enhancing predictive accuracy, the absence of these weights may have contributed to the relatively modest AUCs observed in our analyses.

One of the most important findings of our study is that four risk tools consistently demonstrated higher AUCs when predicting MCI cases that later progressed to dementia, compared to MCI cases that did not particularly for cohorts with long follow-up. This suggests that these risk tools can be tailored to early detection of risk factors for MCI cases who may be at higher risk of developing dementia compare to other MCI cases, which is crucial for implementation of targeted preventive strategies.

The AUCs for predicting MCI were lower than for those predicting dementia using the same tools [11,13]. This is likely due to several factors. MCI is a heterogeneous condition with varied underlying aetiologies and trajectories. The diagnosis of MCI also has low consistency between assessments with a significant number of participants reverting to cognitively normal between waves in cohort studies [29]. This results in higher misclassification of MCI than dementia, making it inherently difficult to predict accurately [30]. Our predictive models focused primarily on modifiable risk factors, with the goal of identifying risks that individuals could potentially address to reduce their dementia risk. While non-modifiable indicators—such as APOE ε4 genotype, results from clinical and neurological examination—may enhance predictive accuracy, their inclusion offers limited value for prevention-oriented strategies [25,31].

The risk tools evaluated here were designed to predict dementia and not MCI specifically. Additionally, our study population consisted primarily of midlife cohorts, aligning with recent evidence highlighting the importance of midlife risk factors in the development of dementia [5]. However, as many MCI cases emerge later in life, the presence and impact of time-varying risk factors (e.g., diabetes, hypertension, stroke, obesity) may differ between midlife and late-life, influencing cognitive outcomes [32,33]. Longer term follow-ups of the cohorts and biomarker confirmation of MCI diagnosis are needed to obtain more accurate evaluation of the sensitivity of dementia risk scores for predicting MCI into late-life. These are also needed to evaluate whether adaptation of dementia risk assessment tools for MCI, or development of MCI specific tools is warranted. A detailed analysis of life-course variation of these risk factors is beyond the scope of the present study, especially given that all of the risk scores evaluated here assumes a constant risk of a risk factors across the life span [34].

One limitation of our study is the definition of cognitive outcome in the Whitehall II dataset. This dataset was previously used in validating a number of dementia risk assessment tools including CAIDE and ANU-ADRI in the literature [35] which shows comparable properties when those tools were validated in other studies [12,36]. We obtained the Whitehall II study dataset from MRC dementia platform UK, and since we are based outside the UK, we were unable to link the dataset with other administrative datasets, such as the UK national Hospital Episode Statistics database, the Mental Health Services Data Set (MHSDS), and the mortality register to boost dementia/MCI diagnosis of the Whitehall study. Despite the weaker outcome measure in the Whitehall II study, the risk tools provided similar performance in this cohort compared with the other cohorts.

Our findings underscore that current dementia risk assessment tools are not only predictive of high risk of dementia in the population [11,36] but also for distinguishing MCI case likely to progress to dementia confirming their overall sensitivity to the spectrum of cognitive impairment. This confirmatory finding supports their use in public health programs to assessment risk and design risk reduction advice. A key strength of these dementia risk prediction tools is that they are all accessible for use in clinical practice, and the more recent tools incorporate more risk factors and provide detailed feedback on risk reduction to assist individuals improve their long term brain health.

## Ethical consideration

Ethical approval for this study was obtained from the University of New South Wales Human Research Ethics Committee. Each of the individual cohorts were approved by their respective institutional review boards.

## Consent to participate

All human subjects provided informed consent to be included in the original studies such as ARIC, Whitehall II and PATH cohort. As we are using secondary data analysis, the consent was not necessary for the analysis.

## Declaration of generative AI and AI-assisted technologies in writing process

No generative AI was used for writing purpose.

## Funding statement

KJA is funded by ARC Laureate Fellowship FL190100011, which also provides support for HH. Huque is also supported by UNSW Ageing Future Institute seed grant (2024).

## Data availability

We have used secondary data analysis. Data may be available from data custodians.

## CRediT authorship contribution statement

**Md Hamidul Huque:** Writing – original draft, Software, Methodology, Formal analysis, Data curation, Conceptualization. **Kaarin J. Anstey:** Writing – review & editing, Supervision, Resources, Funding acquisition, Conceptualization.

## Declaration of competing interest

The authors declare the following financial interests/personal relationships which may be considered as potential competing interests:

Kaarin J. Anstey reports financial support was provided by ARC Laureate Fellowship FL190100011. Md Hamidul Huque reports financial support was provided by 2024 Ageing Future Institute. If there are other authors, they declare that they have no known competing financial interests or personal relationships that could have appeared to influence the work reported in this paper.

## Appendix Supplementary materials

Image, application 1
