# Supplementary material for: Assessment of dementia risk scores in predicting mild cognitive impairment: A comparison of CogDrisk, CAIDE, LIBRA, and ANU-ADRI
Source: J Prev Alzheimers Dis. 2025 Aug 12;12(9):100324. doi: 10.1016/j.tjpad.2025.100324 (PMC12501335; doi:10.1016/j.tjpad.2025.100324)
Supplement: Supplementary file 1 [file mmc1.docx]

Supplementary Table S1: Availability and definition of covariates and outcome in various cohorts

| **Covariates** | **Definition** |
| --- | --- |
| Age of the participants | Age at baseline. The age range at baseline for the ARIC, Whitehall II and PATH datasets are 45-66, 45.1-68.9 and 60-66 years, respectively. |
| Sex | As defined in each cohorts. |
| Education | For the ARIC dataset, education was categorized as: college, graduate school, or professional school as "tertiary"; high school graduate and vocational school as "upper secondary"; and grade school, 0 years of education, or high school without a degree as "less than secondary."  For the Whitehall II and PATH study, education at baseline was categorized based on years of schooling or grades as: "less than secondary" (0-8 years), "upper secondary" (9-11 years), and "tertiary" (12 or more years). |
| Obesity | Obesity was defined based on BMI categorization according to WHO guidelines: underweight (BMI <18.5), normal (BMI 18.5-24.9), overweight (BMI 25-29.9) and obese (BMI >30). |
| Diabetes | For ARIC diabetes variable was calculated based on blood glucose>140mg/dl. For Whitehall II and PATH study self-reported history of diabetes was used. |
| Depression | Defined based on CESD scale in the Whitehall II and ARIC dataset. In PATH dataset, BPHQ scale score >14 is used to define depression. |
| High Cholesterol | Calculated as >6.5 mmol/L or >240 mg/dl n the Whitehall II and ARIC dataset. In PATH, self-reported history of high cholesterol was obtained. |
| Traumatic brain injury | Defined as self-reported history of brain injury / head injury/ knock out with or without consciousness in ARIC and PATH data. Not available in Whitehall study. |
| Smoking | Self-reported smoking status were obtained. |
| Alcohol drink | Self-reported history of alcohol intake with >14 drink/week was defined as high intake, Not requested during Whitehall data acquisition. In PATH, alcohol consumption was calculated according to National Health and Medical Research Council 2001 guidelines[1] using number of drinks per week, with light to moderate intake in males being 0.25–20.5 drinks per week and in females being 0.25–13.5 drinks per week. |
| Loneliness | Not available in ARIC. In the Whitehall and PATH, loneliness was defined based on social network and visits. |
| Physical inactivity | Physical activity was scored as the number of self-reported hours of performing activities at each of three intensity levels: mild, moderate and vigorous activities |
| Cognitive activity | Not available in ARIC and Whitehall datasets. In the PATH study, cognitive activity was calculated based activities on TV/radio, music, ebooks, emails. Games and news. |
| Fish serve | Self-reported fish intake. |
| Hypertension | Hypertension was defined based on self-reported high blood pressure that included both treated and untreated, |
| Stroke | In the ARIC study, a diagnostic computer algorithm was used to define stroke. In the Whitehall clinical diagnosis of stroke was used. In PATH, self-reported history of stroke was used. |
| Coronary heart disease | Self-reported history of CHD was used in ARIC cohort. Not available in Whitehall and PATH dataset. |
| Kidney disease | Kidney disease was defined based on medical history in the ARIC data. Not available in Whitehall and PATH dataset. |
| Atrial Fibrillation | Derived based on MRI data in the ARIC cohort. Not available in Whitehall and PATH dataset. |
| Sleep problem | Defined based on self-reported responses in the Whitehall II study. Not available in ARIC. In PATH sleep problem was defined based on BPHQ 1c. |
| Mild cognitive impairment (MCI) and dementia | In ARIC, MCI and Dementia was diagnosed using an algorithm and ICD-9 codes, following the Diagnostic and Statistical Manual of Mental Disorders (DSM-III-R) criteria[2]. In Whitehall, Probable MCI was defined using MMSE score between 19 and 23[3] and dementia was defined either self-reported history of dementia or MMSE scores<19[4]. In PATH MCI and dementia was diagnosed according to DSM-5 algorithm (for detailed see [5]). |

**References**

[1] Health, N., Council, M.R. Australian alcohol guidelines: health risks and benefits: National Health and Medical Research Council; 2001.

[2] Gottesman, R.F., Albert, M.S., Alonso, A., Coker, L.H., Coresh, J., Davis, S.M., et al. Associations between midlife vascular risk factors and 25-year incident dementia in the Atherosclerosis Risk in Communities (ARIC) cohort. JAMA neurology. 2017;74(10):1246-54.

[3] Kurlowicz, L., Wallace, M. The mini-mental state examination (MMSE). SLACK Incorporated Thorofare, NJ; 1999. p. 8-9.

[4] Folstein, M.F., Folstein, S.E., McHugh, P.R. “Mini-mental state”: a practical method for grading the cognitive state of patients for the clinician. Journal of psychiatric research. 1975;12(3):189-98.

[5] Andrews, S.J., Eramudugolla, R., Velez, J.I., Cherbuin, N., Easteal, S., Anstey, K.J. ANU-ADRI and not Genetic Risk score predicts MCI in a cohort of older adults followed for 12 years. bioRxiv. 2016:070516.
